# Recommendations for the use of long-term experience sampling in bipolar disorder care: a qualitative study of patient and clinician experiences

**DOI:** 10.1186/s40345-020-00201-5

**Published:** 2020-12-01

**Authors:** Fionneke M. Bos, Evelien Snippe, Richard Bruggeman, Bennard Doornbos, Marieke Wichers, Lian van der Krieke

**Affiliations:** 1 Rob Giel Research Center, University of Groningen, University Medical Center Groningen, PO Box 30.001, 9700 RB Groningen, The Netherlands; 2 Interdisciplinary Center Psychopathology and Emotion Regulation (ICPE), University of Groningen, University Medical Center Groningen, Groningen, The Netherlands; 3 Department of Specialized Training, Psychiatric Hospital Mental Health Services Drenthe, Outpatient Clinics, Assen, The Netherlands

**Keywords:** Experience sampling method, Ecological momentary assessment, Bipolar disorder, Self-monitoring, Implementation, Personalized feedback, Qualitative research

## Abstract

**Background:**

Self-monitoring has been shown to improve the self-management and treatment of patients with bipolar disorder. However, current self-monitoring methods are limited to once-daily retrospectively assessed mood, which may not suit the rapid mood fluctuations in bipolar disorder. The experience sampling method (ESM), which assesses mood in real-time several times a day, may overcome these limitations. This study set out to assess the experiences of patients and clinicians with the addition of ESM monitoring, real-time alerts, and personalized feedback to clinical care. Participants were twenty patients with bipolar disorder type I/II and their clinicians. For four months, patients completed five ESM assessments per day on mood, symptoms, and activities. Weekly symptom questionnaires alerted patients and clinicians to potential episodes. After the monitoring, a personalized feedback report based on the patient’s data was discussed between patient and clinician. Three months later, patient and clinician were both interviewed.

**Results:**

Thematic analysis of the transcripts resulted in four themes: perceived effects of the monitoring, alerts, and feedback, and recommendations for implementation of ESM. ESM was perceived as helping patients to cope better with their disorder by increasing awareness, offering new insights, and encouraging life style adjustments. ESM was further believed to facilitate communication between patient and clinician and to lead to new treatment directions. However, high assessment burden and pre-occupation with negative mood and having a disorder were also described. Patients and clinicians advocated for increased personalization and embedding of ESM in care.

**Conclusions:**

This study demonstrates that long-term ESM monitoring, alerts, and personalized feedback are perceived as beneficial to the treatment and self-management of patients with bipolar disorder. Future research should further test the clinical utility of ESM. Clinically relevant feedback and technology need to be developed to enable personalized integration of ESM in clinical care.

## Background

Bipolar disorder is a severe and often lifelong affective disorder, involving depressive and (hypo)manic episodes, and is associated with tremendous burden for patients (Pini et al. 2005; Michalak et al. 2006). Many patients look for ways to successfully live and cope with the impact of the disorder (Lean et al. 2019; Murray et al. 2011). An important strategy to bolster self-management is to self-monitor mood, for example with the NIMH prospective Life-Chart (Leverich and Post 1993; Schärer et al. 2015), ChronoRecord (Bauer et al. 2004), smartphone applications, or rudimentary paper-based methods (Murnane et al. 2016). Indeed, research has suggested that self-monitoring may increase illness insight and self-management, by helping patients to make lifestyle adjustments and facilitate communication with clinicians (Murnane et al. 2016; Bilderbeck et al. 2014). However, existing methods such as the Life-Chart or ChronoRecord have two potential limitations. First, patients have to summarize their symptoms, experiences, and level of functioning into a *single* rating of their overall mood, which might not accurately capture the highly frequent and disparate mood swings patients experience throughout the day (Murnane et al. 2016). Second, the ratings are completed *retrospectively* over the previous 24 h, which may lead to inaccurate information due to mood biases (Reis et al. 2012).

**Fig. 1:**
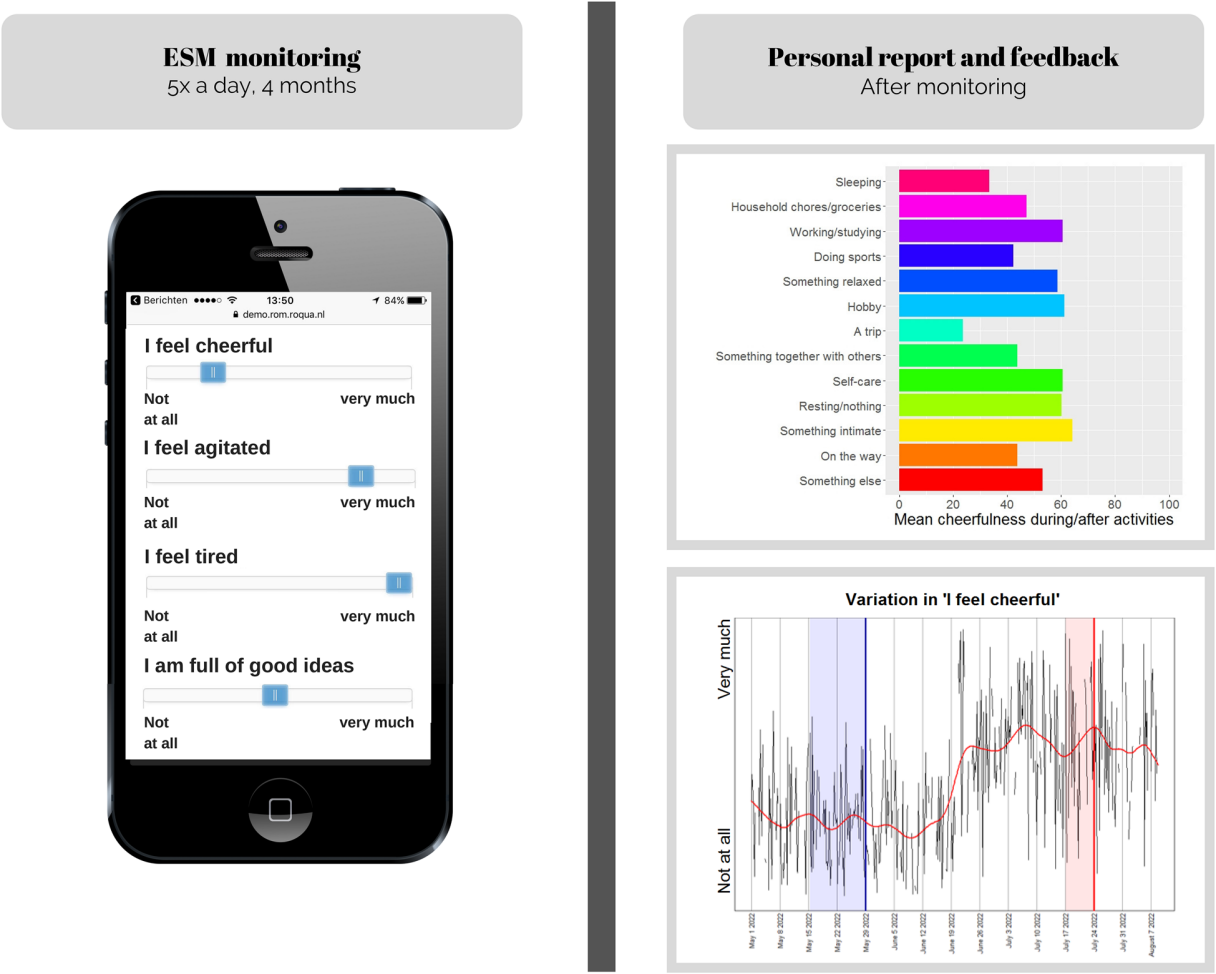
Visual representation of the ESM monitoring and simulated examples of personalized feedback given to participants

The experience sampling method (ESM) is an ecologically valid self-monitoring method that may overcome these shortcomings. Although originally developed for research (Myin-Germeys et al. 2018), ESM has been advocated as a tool to promote self-management and resilience for patients in mental health care (Os et al. 2017; Bos et al. 2019; Wichers et al. 2011). With ESM, patients monitor their daily experiences, moods, and symptoms on their smartphones several times a day (see Fig. 1) (Larson and Csikszentmihalyi 1983). ESM differs from more commonly used self-monitoring methods in that multiple distinct micro-level aspects of mood (e.g., feeling cheerful/down versus manic/depressed) are assessed prospectively, as they occur in daily life. Therefore, ESM may better match patient experiences, and may capture mood fluctuations more accurately (Reis et al. 2012). The detailed ESM data that patients gather can be used to generate personalized feedback on mood variability or associations between mood and lifestyle, or alert patients to impending manic or depressive episodes.

Importantly, patients and clinicians recognize the potential of ESM for clinical care while also noting the possibility of high assessment burden and symptom worsening (Bos et al. 2019). Still, little is known about how patients and clinicians actually experience the addition of ESM to clinical care. This is a crucial first step that will inform on future academic and practical innovations that are currently (technologically) unavailable but will need to be developed for clinical usage. Until now, qualitative studies have focused on feasibility and tolerability (Wenze et al. 2014), evaluating only short-term (1–2 weeks) or low-intensity (1–2 assessments daily) ESM (Wenze et al. 2016; Tsanas et al. 2016), whereas long-term ESM (e.g., several months) may better fit the prolonged nature of bipolar disorder treatments. Furthermore, although personalized feedback seems to be essential for the efficacy of ESM (Kramer et al. 2014), previous qualitative work has been limited to the effects of ESM monitoring (Saunders et al. 2017). Finally, if ESM is to facilitate shared decision making and patient-clinician communication (Bos et al. 2019), the combined experiences of patients and clinicians are needed.

The present qualitative study will therefore comprehensively assess patients’ and clinicians’ experiences with the addition of intensive long-term ESM monitoring, episode alerts, and personalized feedback to clinical care.

## Methods

### Participants

Reporting is done according to the Standards for Reporting Qualitative Research (O’Brien et al. 2014). Participants are twenty patients with bipolar disorder type I or type II receiving treatment, and six clinicians who treated at least two patients participating in this study (see Table 1). Included patients met the following criteria: (1) ≥ 18 years of age, (2) diagnosed with and currently in treatment for bipolar disorder type I/II, and (3) demonstrate high occurrence of episodes (at least 2) in the previous year. Clinicians were selected based on their interest in using ESM in treatment.

**Table 1:** Baseline demographic and clinical characteristics of patients (N = 20) and clinicians (N = 6)

Characteristic	Patients	Clinicians
Gender (N)		
Male	4	5
Female	16	1
Age (N)		
20–35 years	9	0
36–50 years	8	3
51–65 years	3	3
Education level (N)		
Higher education	9	
Secondary education	5	
Secondary vocational education	3	
Pre-vocational education	3	
Years in treatment or years of experience as clinician (*M, SD)*	10.6 (8.8)	16.4 (10.3)
Years since bipolar disorder diagnosis (*M, SD)*	6.4 (6.3)	
Bipolar disorder diagnosis (N) Bipolar disorder type I Bipolar disorder type II	9 11	
Comorbid diagnoses (N) No comorbid Axis I/II disorder Attention Deficit/Hyperactivity Disorder Autism Spectrum Disorder Sleep disorder Alcohol/drug dependence Personality disorder	12 1 1 1 1 6	
Medication use (N) None Amphetamine Anti-epileptic Atypical antipsychotic Benzodiazepine Thyreomimetica Lithium Monoamine oxidase inhibitor Selective serotonin reuptake inhibitor Tricyclic antidepressant	2 1 10 10 9 2 5 3 4 1	
Profession (N)		
Psychiatrist		3
Psychologist		1
Psychiatric nurse		2
Experience with technology in treatment (N)		
None		2
A little experience		4
A lot of experience		0

*Note. ESM* = *experience sampling methodology; M* = *mean; N* = *number; SD* = *standard deviation*

Recruitment took place at two Dutch tertiary care institutions between May 2016 and July 2017. Six clinicians invited their patients to participate in the study until the intended cap of twenty participants was reached. After patients signed up, researchers telephoned them to provide more information. Interested patients were invited to the research facility, where the study was explained in detail.

Clinicians referred 28 patients to the study. Two patients were unreachable. Six patients declined participation during the first telephone call, expecting that study participation and the focus on mood would be too burdensome. This left a total of twenty patients that started and finished the study, of which eighteen were interviewed until data saturation was reached. All participants signed informed consent. The University Medical Center Groningen medical ethics committee approved of the study (201501161).

### Study design

The goal of this project was twofold: to examine whether ESM data can be used to detect early warning signals for mania and depression, and to qualitatively assess experiences with adding ESM to bipolar disorder treatment. The study design was developed to meet both goals.

*ESM.* Patients received five ESM prompts per day for at least four consecutive months. Every three hours (time-contingent schedule), patients received a text message containing a link to the ESM assessment on their smartphone. The assessments were securely administered and stored via RoQua (https://www.roqua.nl) in patients’ personal health records (see Additional file 1). Patients chose their own start and end time and had one hour to complete each assessment (which took approximately 1–2 min to complete). No reminder prompts were given. Researchers contacted patients after the first three days of monitoring and if compliance was low (Baxter and Hunton 2011), if they preferred regular contact, or if the weekly questionnaires (see below) indicated a manic or depressive episode. No financial compensation was offered to participants.

#### Bipolar symptoms

Patients weekly completed the Dutch versions of the Altman Self-Rating Mania Scale (ASRM) (Altman et al. 2001, 1997) and the Quick Inventory for Depressive Symptomatology (QIDS) (Rush et al. 2003; Bernstein et al. 2010). Both the patient and the clinician were e-mailed when scores exceeded cut-offs indicating potential manic (ASRM ≥ 5) (Miller et al. 2009) or depressive (QIDS ≥ 10) (Rush et al. 2003) episodes.

#### ESM items

The questionnaire consisted of 26 Dutch items pertaining to mood (e.g., cheerful, down), symptoms of bipolar disorder (e.g., racing thoughts, feeling inadequate), sleep, and activities (see Additional file 1). These items were based on previous ESM research (Knapen 2019; Krieke et al. 2016; Tyler et al. 2015) and interviews with three patients and a clinician on relevant constructs for people with bipolar disorder. Further, patients formulated one personal item they believed might be insightful to them. Most items were answered on visual analogue scales ranging from 0 (“not at all”) to 100 (“very much”). Patients further had the option to write down anything in a comment field.

#### Compliance

Two patients were part of a pilot and therefore completed three instead of four months of the study. The eighteen other patients all completed at least four months of monitoring with an average of 18 weeks (*range* = 16–32). Near study end, all patients were offered to continue the monitoring for their own benefit (without receiving feedback), which two did for an additional 4 and 14 weeks. Average compliance (number of completed assessments divided by the number of assessments participants received) was 76% (491 assessments, *SD* = 137.8).

### ESM feedback

Within one month after the end of the ESM monitoring, researcher FB constructed a personal feedback report (see Additional files 1, 2). This report contained information on (1) variation in mood and symptoms in general and related to time of day and activities, (2) frequency of activities, (3) occurrence of episodes in association with ESM items, (4) sleeping patterns, (5) personal questions, and (6) comment field entries. The feedback session took place on average 68 days (*SD* = 33.0) after the monitoring period had ended. Clinicians explained the feedback, asked patients to interpret unexpected findings, and helped them formulate conclusions. All clinicians were briefed beforehand on the interpretation of the ESM feedback and researcher FB was present to provide explanations if necessary. If new questions arose (in five cases), the researcher ran additional analyses and e-mailed the results to both patient and clinician.

### Interviews

Follow-up interviews were held with 18 patients and 6 clinicians and took place at the research facility between April 2017 and March 2018. The interviews were planned three months after the feedback session (*M* = 107 days, *SD* = 21.5) and had an average duration of 46.8 min. Participants were explained the goal of the interview: to learn more about their experiences with adding ESM to clinical care. The interview started with the open question how participants reflected on their experiences with ESM. After fully exploring their first responses to this question, a semi-structured interview guide was used to ask open questions on (1) experiences with the ESM monitoring, the alerts, and the report and feedback session, (2) potential insights gained, (3) potential behavioral changes made, and (4) the utility of ESM for clinical care. Further, person-specific questions were asked using field notes made during the monitoring phase and the feedback session. All interviews were audio-recorded and transcribed verbatim. Interviews were conducted by FB (M.Sc., female, PhD-student) and ES (Ph.D., female, postdoctoral researcher), both trained in qualitative interviewing and analysis. At the time of the interview, all participants knew FB from the introduction and follow-up calls.

### Qualitative data analysis

To understand patients’ and clinicians’ experiences with adding ESM to clinical care, thematic analysis was applied on the transcripts by FB, LK, and ES according to the Qualitative analysis Guide of Leuven (Casterle et al. 2012). Field notes and observations were analyzed to understand the effects of ESM monitoring while it took place.

First, transcripts were summarized in conceptual interview schemes and narrative reports to gain a holistic understanding of participants’ experiences. Based on subthemes identified in the data (e.g., awareness), a concept code list was constructed. This code list was then used to code the transcripts in Atlas.TI (version 8), creating new codes when previously unidentified themes were encountered. The codes were then grouped in four central themes, which were verified against all transcripts and discussed with all authors. Participants were invited to provide feedback on a summary of the themes.

## Results

**Fig. 2:**
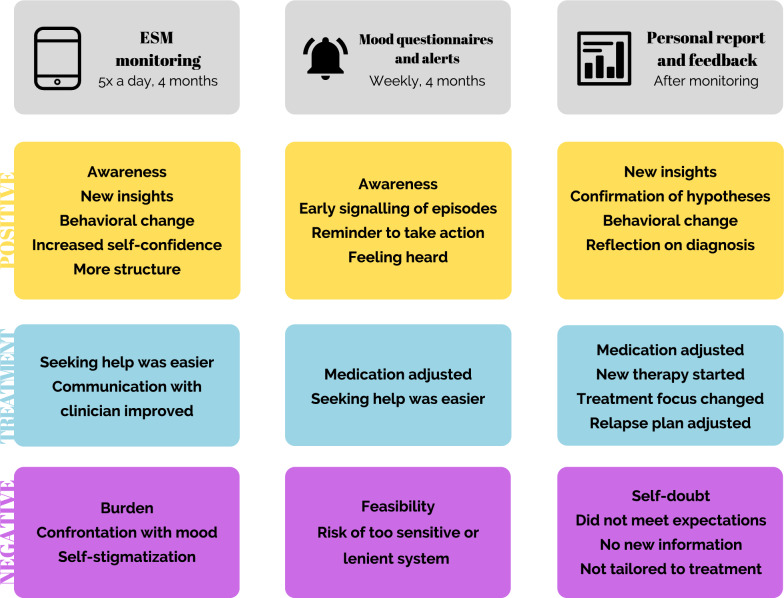
Schematic overview of the main findings of the first three themes: perceived effects of ESM monitoring, the weekly questionnaires (ASRM/QIDS) and alerts, and the personal report and feedback session

Thematic analysis resulted in four themes (see Fig. 2): (1) effects of ESM monitoring, (2) effects of the weekly symptom questionnaires and alerts, (3) effects of the personal report and feedback session, and (4) recommendations on the use of ESM in clinical practice. For each aspect of ESM, patients and clinicians described perceived positive and negative effects, and effects on treatment they attributed to ESM. See Tables 2 and 3 for illustrative quotes related to the themes.

**Table 2:** Quotes related to theme 1 (effects of monitoring) and theme 2 (effects of weekly questionnaires and alerts)

Quote patients	Quote clinicians
Theme 1: effects of monitoring	
ID13: During the monitoring period I have mentioned this several times, but what really surprised me and helped me a lot was the compartmentalization in those five parts a day. That really was a revelation that I had never heard before in mental health care. Nobody had divided it in small pieces of three hours. Previously, I only had the Life Chart, once every 24 h. A big yes, or a big no, or a big wow or a big ‘bleh’. And now, something unpleasant could happen, and it would make sense that it makes me feel bad, or hyper, or sad. That will maybe last a part of three hours, but then my mood is… […] So this is what I learned from the monitoring, and I don’t know if this was the intended effect of the study. But what I learned is to look at myself much more objectively, and much more relaxed. (female patient in her forties)	ID22: Yes, that they become much more aware of factors influencing their mood. And that really differs across persons. That’s one thing. Or the fact that they become much more aware of their vulnerability in developing mood swings. An important part of treatment is about accepting that you are chronically instable. Some people keep wanting a sort of stable phase or that everything will be okay again, will return to how it was before. And that of course doesn’t always work out like that. And this [ESM] holds up some sort of mirror for them, of course. (male psychiatrist in his thirties)
ID2: In the period that I filled in the assessments, I experienced several times that I answered that I hadn’t been outside, for example, during a week I was at my mothers’ place sitting around and being depressed. And then I had to answer three times that I hadn’t yet been outside, and no, I didn’t feel that well. You know, like that, and then I thought, well, okay I’ll just go. That happened multiple times I think. (female patient in her twenties)	ID25: Yes, this really helps, you get to the point much more easily, and can give better targeted lifestyle advice. If they haven’t already developed those insights themselves. That is what I believe to be the advantage of self-monitoring and assignments you can do at home, outside our conversations here in the clinic: that you can adapt your own behavior and make healthy choices, so that is a nice side effect of this study, I think. (male psychiatric nurse in his fifties)
ID17: Especially when the assessments come at an inconvenient moment. I find that very stressful. That is mostly the problem. The questions were completed in no time, but just, when I was in the car for example, a text comes, and I keep thinking, “I should not forget, I should not forget”. That’s it. No, the amount of work itself was not that much. (female patient in her forties)	ID24: On the Life Chart you can indicate that you score this or that, on average. A lot of people will then say that the actual situation is very different. So the micro-level is much more fine-grained. The danger is, though, that if people feel very bad, because their relationship has ended or I don’t know, that they will immediately think that they have a depression. That the micro-level overshadows the macro-level. (male psychiatrist in his sixties)
ID8: By continually confronting you with it, you keep getting reminded of the fact you’re doing badly. Or badly… Sad, that you’re feeling sad. So then I found it hard to look at it another way. Because normally, I do that, I try to do things differently and find distraction and everything. But when I looked for distractions, I got a new assessment, making me think, “damn, I am indeed doing very badly”. And that’s what I found really annoying, or really annoying… I didn’t like that. (female patient in her thirties)	ID23: Well, if there are people who keep getting hung up on it and keep feeling sad as a result, then I find that a negative consequence. But still, if that is the case, it suggests to me that we [patient and clinician] have to work on that. So in that sense, it can be helpful. (male psychiatrist in his forties)
ID15: Now I’m doing well, and I complete the Life Chart every morning. And then, for the rest of the day, I don’t have to think about my having bipolar disorder. Because then I know that I’m okay, I don’t have to pay attention to anything. But if you have to complete a questionnaire five times a day, then you really get confronted five times a day that you have that disorder. Throughout the day, you keep being confronted with ‘you have a disorder’. (female patient in her fifties)	Interviewer: Was the burden too much? Weighing it against what patients gained from it? ID23: Maybe. Maybe it was too much, but you don’t know that beforehand. That’s why I think: you have to try. And self-management is a major step. So to invest a good amount of energy into that, because you have a severe disorder, you can invest a lot of energy into that, and then it is actually helpful to have something like this available to see if it gives you more insight. So in hindsight, yes it might have been burdensome, but I find that a bit too easy. Although, if you start using it now as a tool in clinical practice, then it might have to be toned down just a little. (male psychiatrist in his forties)
Theme 2: effects of weekly questionnaires and alerts	
ID14: I had expected that, when feeling more manic, or hypomanic, I would really find the questionnaire stupid. That’s what I expected, but that happened actually right near the end of the ESM monitoring period, that I noticed “something is happening and I don’t really trust it”. And you also notified me of elevated things. And at that moment, that was actually really nice. Like, I really have to take step back. I feel fantastic, that’s not it, but I hadn’t realized yet that the scores were high until I saw it in the questionnaires. And then I could admit it more easily to myself, that maybe I had to take a step back. I will e-mail [clinician]. That was a really good experience that really helped me. Like: if I see it coming beforehand one way or another, because usually I notice it too late, then I experience everything less intense. (female patient in her twenties)	ID22: Well, I was really busy then, and then I also got those alerts and I thought: “do I have to do something with this as well?” That felt a bit as a responsibility, in a way. Whereas I always believe, you know, people really have to reach out themselves. That’s what you teach them, that we don’t take it all over and take care of them. So you really need to make clear agreements beforehand, like “what are we going to do when I see this?” And now, it just happened. I think it is something you can use in your treatment, but then you really have to discuss with patients, “what will we do, do you want me to reach out, or not? You get the alerts, do you appreciate that or not?” I think that is a good opportunity, but you have to think about this really well.” (male psychiatrist in his thirties)
ID7: That I was not alone or let go in this. Because on the one hand, I am really inclined to go my own way and withdraw myself, really disregard everything and everyone. But back then I would consistently complete the questionnaires. And well, that by doing so I was not and could not be invisible. And actually, I like that. Because the withdrawing that I do, I actually don’t want to do that. And then it helps if somewhere a graph shows: “this woman is not doing well. And I will e-mail her.” (female patient in her fifties)

**Table 3:** Quotes related to theme 3 (effects of personal report and feedback session) and theme 4 (recommendations on the use of ESM in clinical practice)

Quote patients	Quote clinicians
Theme 3: effects of personal report	
ID2: Actually, last week a lot of people asked me to go here or go there, and I told them, “actually I’m not feeling so well, I’m not sure.” And they started saying, “what does it matter, if you’re already sick you should come anyway and be sick tomorrow”. And then I thought, “no, I’m not doing this because I know how this will go, then it will happen that day and again and again the next days and before you know it, I’m really not doing well and that will have its effect on others as well.” So in that way it worked maybe, a sort of small life lessons. Interviewer: Is that something that the report taught you, or? ID2: Well, to see that on paper, that really worked, those large mood swings. That you really have some sort of reflective moment. This is what happened then, and in that sense I think unconsciously shaped the way I think, I think. (female patient in her twenties)	ID25: For example, for one client, you later explicitly investigated sleeping, tiredness. After using cannabis, for example, the night before. Well, those very specific data coming out of the study are very helpful. Because I can have a very strong intuition that something is the case, but now we have it on paper, it is confirmed. Because she herself has supplied the data that shed a light on the situation. And there were more explicit outcomes: hours of sleep, energy, that’s what we discussed, that’s helpful to integrate in relapse prevention plans. (male psychiatric nurse in his fifties)
ID15: Now I know immediately that when I really start to worry and feel tense, it is the beginning of a depressive episode. If it lasts for about a week, I know that it is the start of a depressive episode, and I start taking medications, and I don’t sink so deeply. Interviewer: Does this help? ID15: Yes, because I start taking my medications sooner. Because usually it takes a week or three before they start working, and then I notice a bit sooner that the tension starts to disappear. Normally I am really tense first and I no longer want anything at all, and because the medications take a while to start working, you reach the point that nothing works anymore, that you really have to fight to keep doing your daily activities. Now I get there on time, because I’ve already started taking my medications. So I don’t sink so deeply anymore. (female patient in her fifties)	
ID14: Ah yes, I couldn’t do anything with it [personal report]. But that maybe also has something to do with my expectations. I don’t know what I’d expected. Probably I’d expected that I… that something about myself I didn’t … That’s what I’d hoped, maybe. I’d hoped that something would come out that would help. A piece of the puzzle. You know. You really want that it does something big. And it mostly was a confirmation of everything I already knew. And that is okay, but that is not what I’d hoped. Nice that I know myself better than I thought, I liked that, that there were no surprises. But I also thought, “and what now, now I have this, and what should I do?” So it didn’t help me as much. (female patient in her twenties)	ID23: If you look at the results, it really is so hard to interpret them. It’s still much more complicated than you had hoped beforehand. On the one hand, it’s a lot of data and I like graphs and such, I think they’re nice, you have a sort of overview, and well, about activities and such, it is solid. But what comes out as predictors disappoints me. Such that I think: it’s not so unequivocal or it’s not so easy to predict. Especially for people who are so instable in their mood, then the story gets even more unclear. (male psychiatrist in his forties)
Theme 4: recommendations for the use of ESM in clinical practice	
ID5: Like I’m saying, if you’re young you’re really inclined to go against everything. If somebody says something, you won’t accept it, whereas if you’ve experienced it yourself, then you just know, you can’t go around it. You see, without such a study it can take years before you’ve been through all that or have experienced a relapse or episode. And that’s such a pity. Whereas if you can demonstrate such small changes with this study, they don’t have to experience it all themselves. That they don’t experience all the very heavy consequences, but see the small changes in themselves, which they have filled out themselves. (female patient in her forties)	ID25: I would really try to develop it tailored to the situation of the patient. And maybe link it to the relapse prevention plan. And it would be even better to also link it to the Life Chart method, for example. Or a sort of mood app, right? I mean, those exist, but are usually not so comprehensive. This way, you have all the information that you could use in treatment, and you have the aspect of self-management that can directly, in that moment, be adapted or stimulated even. (male psychiatric nurse in his fifties)
ID10: During therapy or something, it [ESM] might also be very easy. Then the system could directly inform your clinician, rather than bringing a copy yourself, so to speak. That they [the clinicians] could directly, if you give your consent, have insight in the data. And yes, the system doesn’t lie. You can show that you have filled it out, at those moments. (male patient in his twenties)	ID26: The difficulty remains that this is a self-report measure, so people indicate their own visions on their problems. There are people, if you ask them a number between 1 and 10 to indicate their stress level, who will say a 10 with a very calm demeanor. Or the other way around, sitting there like thís [raising arms to indicate high stress level] saying, it’s a 5. An app like this [ESM] will have it wrong too, people are not so good at judging themselves. So you always have to be aware that it is not a science, in fact it is their vision on their problems. (female psychologist in her forties)

### Effects of ESM monitoring

#### Positive effects

First, almost all patients described increased awareness of their mood and symptoms, behavior, well-being, factors influencing mood, and positive aspects in their lives. Many patients contrasted the Life-Chart with ESM and highlighted the benefits of dividing their mood into multiple distinct components (e.g., agitated, easily distracted, racing thoughts) rather than focusing on mania or depression as a whole, making it easier to judge their mood and the likelihood of impending episodes. Several patients described heightened mood awareness even after the monitoring period was over. Clinicians confirmed seeing heightened awareness in their patients. Both patients and clinicians found this one of the most helpful aspects of ESM monitoring.

Second, many patients described gaining new insights on the impact of their behavior, activities, events, and time of day on their mood, which their clinicians confirmed. Other patients realized that their mood could also act independently of their behavior or context, which made mood swings easier to accept. Two patients learned that their mood fluctuated much more during the day than expected. Two other patients learned they had trouble recognizing their emotions, and felt that this improved during the monitoring period. One patient learned that her job was too heavy for her. One clinician gained new insights into his patients’ coping strategies. Many patients and clinicians indicated that ESM monitoring helped patients discover these insights by themselves, thereby firmly consolidating them.

Third, more than half of the patients made concrete behavioral changes to influence mood and symptoms (e.g., becoming more active when feeling depressed, or slowing down when experiencing (hypo)mania). Clinicians confirmed this observation. Several patients continued monitoring their mood in a diary or the Life-Chart. One patient felt she could safely experiment with the strict behavioral rules she had learned during years of therapy and investigated the effects with the ESM monitoring. One patient described that the monitoring led to more meaningful conversations with her environment about what it means to have bipolar disorder.

Finally, a few patients described gaining more self-confidence in rating their mood and becoming more structured due to the steady rhythm of the assessments each day.

#### Effects on treatment

Some patients felt that the threshold to seek help was lowered, and that their increased awareness helped in the communication with their clinician.

#### Negative effects

Patients attributed several negative effects to the ESM monitoring. First, although many patients noted that the assessments were quick and easy to complete, most patients found five assessments per day burdensome because they felt disturbed in daily activities. Some felt they constantly kept (the possibility of) assessments in their minds and felt guilty or irritated when missing them. Nonetheless, most patients indicated that the benefits of the monitoring outweighed the burden, which aligned with the impression of their clinicians.

Secondly, approximately half of the patients felt negatively influenced by the frequent confrontation with their moods and symptoms, especially when already feeling depressed or (hypo)manic. When feeling depressed, several patients described that this confrontation made them feel restless, emotional, or irritated, and made them worry about their wellbeing. Alternatively, when experiencing (hypo)mania, several patients felt too busy and wanted to avoid reflection when feeling good. Of those describing a negative response, around half added that the confrontation actually helped them to become aware of their mood and either accept it, act upon it, or put it into perspective. The other half believed the assessments impaired their usual coping strategy, seeking distraction. Several clinicians agreed that confrontation might be difficult for patients, although increased awareness might mobilize patients into action.

Finally, a few patients felt that the assessments were a constant reminder of their diagnosis, the “unhealthy” part of themselves. They described that each assessment reminded them that they monitored themselves because of their bipolar disorder. In this way, ESM monitoring was perceived as self-stigmatizing, which two clinicians also warned against.

### Effects of the weekly questionnaires and alerts

#### Positive effects

First, several patients described that the alerts made them more aware of their mood and events of the past week, and possible ways to cope when mood worsened. For one patient, the alerts signaled a hypomanic state before realizing it herself, allowing her to get early treatment for the first time. Several others found that the alerts confirmed what they or their environment already suspected, which helped coax them into action. Second, many patients felt supported by the idea that someone (here, the researcher) would inform them of elevated symptoms. They described feeling heard and less alone.

#### Effects on treatment

For one patient, medication was occasionally adjusted upon receiving alerts. Several other patients found it easier to seek help and contacted their clinician. Clinicians differed in whether they contacted patients themselves or let patients seek contact and argued that this might differ across patients. The rationale given for not contacting patients was that recognizing and acting upon episodes are important treatment goals, and patients should be encouraged to seek help themselves. As such, several clinicians emphasized the need for making clear agreements with patients on who contacts whom in case of elevated scores. Clinicians also questioned the feasibility of acting upon alerts if sent for their entire caseload.

#### Negative effects

Patients did not describe negative effects. Two patients were curious of (but not bothered by) the timing of the alerts; they either received alerts when feeling well, or did not receive alerts when they signaled episodes themselves (e.g., QIDS score exceeded cut-off but for less than three weeks). Some clinicians suggested that a too sensitive alert system could lead to panic or demotivate patients.

### Effects of the personal report and feedback session

Many patients and clinicians were positively surprised by the amounts of data collected by patients. The report was described as ‘mapping yourself’, and ‘a personal history on a very small scale’. Many patients reread the report at home or showed it to close family or friends.

#### Positive effects

First, many patients described that the report helped them in gaining new insights on (1) the association between mood, behavior, sleep, and activities, (2) the presence or duration of emotions, (3) the benefits of healthy life style adjustments, (4) warning signals for manic or depressive episodes, and (5) the frequency and duration of behaviors and activities. Two patients used the personal report to specifically test ideas about themselves or their life style (e.g., effects of soft drugs on mood), which they saw confirmed. Many patients explicitly mentioned that the report was a more accurate, objective, and detailed reflection of their wellbeing during the monitoring period than their own account, which they described to be often discolored by present mood. Although clinicians did not describe new insights themselves, they believed that the report had been insightful to patients.

Second, several patients attributed lasting behavioral changes to the report. Two patients noticed the effects of sleep on their wellbeing, and started slowing down and cancelling appointments when they slept badly. Others learned how activities affected their mood, and started doing positive activities more often, even when feeling depressed. Finally, one patient concluded from the report that feeling tense preceded depressive episodes, and started taking medication when noticing tension. At the time of the interview, this had occurred several times, and she felt that this strategy had made the episodes less intense. Many patients felt that the graphs objectively showed the benefits of certain behavioral changes in their own data, making it easier to accept and act on these insights. The visual nature of the report further helped some patients to ‘get out of their head’.

Finally, the personal report made several patients reflect on their diagnosis: some described that it was helpful to once again realize and accept the impact of the disorder, which was also described by two clinicians. Although a few other patients had hoped that the report would give rise to a different diagnosis, they found it a helpful reminder that sometimes, they could feel bad for no apparent reason.

#### Effects on treatment

Five patients and their clinicians made changes in treatment that they attributed to the personal report. One patient started emotion regulation therapy based on her typed entries. For one patient, the relapse prevention plan was adjusted based on new insights in the personal report. For another patient, the focus of treatment shifted, because the report had shown that the previous focus was no longer relevant. Yet another patient was referred to a psychologist. Finally, medication was adjusted for one patient. Several other patients and clinicians mentioned that the report provided them with a helpful framework to discuss the course of treatment and the patient-clinician relationship. Several patients and clinicians had planned to adjust the relapse plan or refer the patient, but had not yet started the process.

#### Negative effects

A few negative effects were described. Some patients were apprehensive of the feedback session, thinking that the results might have implications for their diagnosis or treatment. One patient, whose data showed large mood fluctuations, started doubting her diagnosis and ability to judge her mood: contrary to her own experiences, she had inadvertently understood from the report that she had experienced no manic episodes.

Although many patients and clinicians mostly reflected positively on the personal report, they were also somewhat disappointed by it. Many had high expectations, believing the report would suggest novel directions for treatment, indicate a different diagnosis, or provide the missing piece of the puzzle to help patients cope better with their complaints. They felt that the descriptive nature of the report did not meet their need for unambiguous conclusions. Some clinicians noted that so many factors appeared to influence mood and symptoms, that it was hard to find clear patterns in ESM data, especially because most patients showed erratic mood fluctuations. Some patients felt that the report only confirmed what they already knew or had learned during the monitoring period, or were unhappy that the monitoring took place during an unrepresentative period (e.g., during holidays or stable periods). A few patients further found the graphs difficult to understand.

Several clinicians added that the report was difficult to tie to treatment goals, and that it might have been more effective to discuss the feedback more frequently than just at the end of the monitoring period. Finally, two clinicians warned that ESM results could falsely suggest a hard truth, while ESM data supposedly only shows the patient’s vision on their complaints. They stressed the need for clinicians to explain this to patients.

### Recommendations for the use of ESM in clinical care

Based on their experiences with ESM, patients and clinicians reflected on the use of ESM in clinical care.

#### For whom and when

Many patients and clinicians would especially recommend ESM for young, recently diagnosed people, for whom the insights yielded by ESM are still new. Clinicians also proposed patients in long-term care who keep having trouble and need a fresh perspective. Some patients and clinicians would refrain from ESM during (1) highly unstable or bad periods, because ESM could be too confrontational, (2) prolonged (depressive) periods without change, (3) good periods, when patients do not want to worry about their diagnosis, and 4) periods when there is insufficient suffering to motivate patients for ESM. Indeed, all but two patients opted not to continue the monitoring after four months, when they believed to have gained sufficient insights and additional data gathering was no longer necessary for the personal report. Several clinicians would not recommend ESM to patients already very pre-occupied with their disorder.

#### Goals

The majority of patients and clinicians advocated that ESM be tailored to the patient’s situation and treatment goal. Many patients would consider doing ESM again when (1) symptoms increase, (2) specific hypotheses arise, (3) starting new medications or treatments, or (4) they want to monitor for episodes. Clinicians highlighted the potential of providing alerts and advice in case of elevated ESM scores, to train patients in recognizing mood changes and getting help on time. Two clinicians deemed ESM more reliable and insightful than commonly used methods like paper-and-pencil registration or the Life-Chart (5).

#### ESM monitoring

Many patients and clinicians would have preferred the possibility to personalize the content, frequency, and duration of the ESM diary to better fit the patient’s situation and treatment goal. Furthermore, many patients and clinicians liked the combination between micro-level ESM and macro-level symptom questionnaires.

#### Data access

Many patients and clinicians suggested discussing ESM feedback at every treatment session, to keep the feedback relevant and the patient motivated. Some patients would have liked to examine the feedback in between sessions. Others found an overview feedback report after several months more relevant. Some patients suggested that patients should be offered the choice to share their data with their clinicians, whereas most patients assumed that all data is automatically available to both themselves and their clinician.

#### Report

On top of the information included in the report, several patients and clinicians would like to have seen (1) effects of starting or stopping medication or treatment, (2) effects of life style adjustments, (3) testing of specific hypotheses, (4) decisions on diagnosis, or (5) simple explanations of the data analysis.

#### Clinician role

All patients and clinicians saw an important role for the clinician in the process of ESM, especially in discussing the personal feedback. Many patients preferred this clinician to be someone they know well and see frequently. Patients and clinicians further emphasized that clinicians should learn how to interpret ESM feedback and should believe in the potential of ESM. Patients and clinicians suggested the feedback session is successful if the clinician (1) invests time to agree on the goal, content, and interpretation of ESM, (2) facilitates conversation on the meaning of the results, (3) teaches patients how to interpret ESM feedback, (4) is conscious of potential negative effects, and (5) makes clear agreements on what to do in case of elevated scores.

## Discussion

The present qualitative study examined how patients with bipolar disorder and their clinicians experienced the addition of long-term ESM (i.e., 4 months, 5 assessments daily), episode alerts, and personalized feedback to clinical care. Confirming other qualitative work (Saunders et al. 2017), most patients and clinicians reported that ESM monitoring and personalized feedback helped patients to cope better with their disorder by increasing awareness, offering new insights, and encouraging life style adjustments. In addition, as has been described as the promise of ESM (Murnane et al. 2016; Bos et al. 2019; Saunders et al. 2017), the monitoring and alerts were perceived as facilitating communication between patient and clinician. A relatively small group perceived the personalized feedback as helpful in informing treatment directions. As such, the present study is the first to demonstrate that long-term ESM is perceived to be a helpful tool for patients with bipolar disorder and their clinicians.

The present qualitative findings warrant further quantitative research to confirm both the positive and negative effects of adding ESM to bipolar disorder treatment. Potential beneficial effects of ESM should be weighed against the considerable burden and the risk of negative effects reported by several patients, namely the confrontation with (negative) mood and pre-occupation with having a disorder. Indeed, even though quantitative studies often report high compliance rates (Vachon et al. 2019), qualitative work consistently demonstrates that patients and clinicians can be apprehensive of these effects (Bos et al. 2019; Saunders et al. 2017). For some patients, this may be sufficient reason not to participate in ESM. ESM seems to carry the risk of making a small yet significant group of patients hyperaware of their mood, especially during mood episodes (Genugten et al. 2020; Conner and Reid 2012), thereby cutting off adaptive coping strategies such as seeking distraction (Schermer 2009). Therefore, when implementing ESM, clinicians have to be aware of potential negative effects and discuss them with patients, whereas researchers have to ensure that ESM assessments minimize mood reactivity (Myin-Germeys 2012).

Notable was the finding that the descriptive nature of the personal feedback report disappointed patients and clinicians, who had hoped for more clear-cut advice on diagnosis and ways to improve well-being. Statistical analyses currently under development show promise in summarizing complex ESM data by predicting diagnosis (Perez Arribas et al. 2018), forecasting episodes (Smit et al. 2019), or testing associations (Snippe et al. 2016). These techniques could also inform on a personalized alert system that optimizes both sensitivity and specificity. However, these analyses are still relatively new and need empirical testing before they can be implemented in individual patients (Wichers et al. 2019; Faurholt-Jepsen et al. 2018). A recent study reported that, even when the same data was used, different analytical approaches led to highly disparate clinical advice across research groups (Bastiaansen et al. 2020). Indeed, patients in our sample attributed high credibility to scientific data even when it contradicted their own beliefs. These results urge researchers to further develop valid models. Until they become available, researchers should carefully manage expectations of patients and clinicians on the possibilities of ESM feedback.

In the present study, the ESM diary, alerts, and personalized feedback were standardized and minimally embedded in clinical care. However, patients and clinicians indicated a desire for more personalization and integration in care, as these ESM components can be expected to differ across people, and even vary within patients, based on current treatment goals and the patient situation (Bos et al. 2019). This is further exemplified by the finding that only 2 out of 20 patients continued monitoring when feedback and embedding in care were no longer provided. For the academic field, this means considerable effort needs to be invested into developing the necessary technology for the integration of personalized ESM in clinical care. This translation from research to practice is often overlooked (Os et al. 2013; Fisher and Boswell 2016) but imperative for its usability. Integration requires a user-friendly interface that helps patients and clinicians to construct a personalized and scientifically valid ESM diary, sets sensitive alerts if needed, and provides them with automatically available personalized feedback. Such an endeavor necessitates intensive collaboration between researchers, patients, clinicians, and software developers. Currently, our research team is developing such a tool termed PETRA (PErsonalized Treatment by Real-Time Assessment, https://www.petrapsy.nl/en/).

Finally, if ESM should become available to clinical care, the question remains if, when, and how ESM can be useful and feasible for any given patient. For example, the low-intensity yearlong self-monitoring with the Life-Chart (Leverich and Post 1993) or ChronoRecord (Bauer et al. 2004) might suffice for some, whereas for others, the high-intensity and detailed ESM might better fit their needs. The sampling schedule will greatly depend on the intended goal of the self-monitoring: e.g., enhancing self-management, tracking treatment effects, or informing diagnostics. Therefore, many participants recommended involving both patient and clinician equally in all decisions regarding ESM: the goal and desirability of ESM, potential negative effects and burden, the ESM diary content and schedule, clinician involvement, and ESM feedback interpretation. Reaching agreement on these points beforehand is essential for enhancing patient self-management and empowerment through self-monitoring technology (Schermer 2009). Based on their experiences, we have formulated crucial issues for patients and clinicians to discuss together when considering using ESM (see Table 4). These discussion points can help in deciding if and how to use ESM in treatment.

**Table 4:** Practical discussion points for clinicians and patients to consider before starting ESM in treatment, based on the findings of the present study

1: Determine rationale of ESM	
Desirability of ESM	Do both patient and clinician agree that ESM is helpful and doable?
Goal of ESM	What do patient and clinician hope to gain from ESM? How does it fit into the patients’ current treatment goals?
2: Manage expectations	
Risk of negative effects	Are patient or clinician apprehensive of any negative effects (e.g., mood worsening, pre-occupation with disorder)? What can the patient do if these occur?
Burden	Is it okay if patients’ occasionally miss assessments, and how often?
Feedback	What can patient and clinician expect to learn from the ESM feedback, and what not?
3: Determine the ESM protocol	
Feasibility of the monitoring	What frequency and duration of assessments is necessary to meet the goal and remain feasible for both patient and clinician?
Content of the assessments	What should the ESM diary include to meet the goal?
Need for weekly mood questionnaires	Is weekly monitoring for episodes necessary?
Desirability of alerts to patient and/or clinician	Do patient and clinician want to be informed of elevated scores?
4: Determine level of involvement of clinician	
Data access	What data is the clinician allowed to examine and how often?
Degree of contact through ESM	Does the patient contact the clinician in case of elevated scores or alerts, or vice versa? What happens if patients indicate elevated scores?
5: Facilitate interpretation of personalized feedback	
Frequency of feedback	How often is the personalized feedback discussed?
Content of feedback	Which data are discussed? Is it necessary to discuss all the feedback every session, or only parts of it?
Interpretation of feedback	Can the clinician help the patient to read the graphs? How do both interpret the feedback, and are there meaningful differences therein?
6: Evaluate regularly	
Usefulness and feasibility of ESM	Does the current ESM protocol still meet its intended goal or does it needs to be adapted? Is it still feasible for the patient?
Negative effects	Have any negative effects of ESM occurred and (how) can the patient cope with them?

Strengths of our study include the prolonged ESM monitoring period, which so far has not been reported elsewhere, and our in-depth exploration of patients’ and clinicians’ experiences with all aspects of ESM (long-term monitoring, alerts, and personalized feedback). Their suggestions can inform future academic and clinical endeavors on the utility of ESM and patients and clinicians willing to experiment with ESM.

A limitation of this study is its convenience sample: 80% of included patients were women, relatively few clinicians were included, and participants were selected (in part) on their interest in self-monitoring. Given our sample of convenience, as well as the fast-moving field and the diversity in healthcare systems, generalizations to other settings should be done cautiously. Additionally, any positive or negative effects that patients and clinicians attributed to ESM could be due to other causes unknown to them. Finally, clinicians were less involved during the monitoring period, which limited integration of ESM in treatment.

## Conclusions

This study provides demonstrates that long-term ESM monitoring, alerts, and personalized feedback are perceived as beneficial to the treatment and self-management of patients with bipolar disorder. To optimize clinical utility of ESM, our results suggest that future research should prioritize the development of clinically relevant data analyses and the necessary technology to enable personalized integration of ESM in clinical care. Furthermore, we recommend intensive collaboration between patient and clinician to ensure ESM fits the patient’s situation and treatment goals. This way, ESM has the potential improve clinical care.

## Supplementary information

**Additional file 1.** ESM items, data security, and example of feedback report.

**Additional file 2.** R code for the feedback report.

## Data Availability

The qualitative interview data used and analyzed during the current study are available from the corresponding author on reasonable request.

## References

[CR1] Altman EG, Hedeker D, Peterson JL, Davis JM (1997). The Altman self-rating mania scale. Biol Psychiatry.

[CR2] Altman E, Hedeker D, Peterson JL, Davis JM (2001). A comparative evaluation of three self-rating scales for acute mania. BiolPsychiatry.

[CR3] Bastiaansen JA, Kunkels YK, Blaauw FJ, Boker SM, Ceulemans E, Chen M (2020). Time to get personal? The impact of researchers choices on the selection of treatment targets using the experience sampling methodology. J Psychosom Res..

[CR4] Bauer M, Grof P, Gyulai L, Rasgon N, Glenn T, Whybrow PC (2004). Using technology to improve longitudinal studies: self-reporting with ChronoRecord in bipolar disorder. Bipolar Disord.

[CR5] Baxter RJ, Hunton JE (2011). Capturing affect via the experience sampling method: prospects for accounting information systems researchers. Int J Account Inform Syst.

[CR6] Bernstein IH, Rush AJ, Suppes T, Kyotoku Y, Warden D (2010). The Quick Inventory of Depressive Symptomatology (clinician and self-report versions) in patients with bipolar disorder. CNS Spectr.

[CR7] Bilderbeck AC, Saunders KE, Price J, Goodwin GM (2014). Psychiatric assessment of mood instability: qualitative study of patient experience. Br J Psychiatry.

[CR8] Bos FM, Snippe E, Bruggeman R, Wichers M, van der Krieke L (2019). Insights of patients and clinicians on the promise of the experience sampling method for psychiatric care. Psychiatr Serv.

[CR9] Conner TS, Reid KA (2012). Effects of intensive mobile happiness reporting in daily life. Soc Psychol Person Sci.

[CR10] de Casterle BD, Gastmans C, Bryon E, Denier Y (2012). QUAGOL: A guide for qualitative data analysis. Int J Nurs Stud.

[CR11] Faurholt-Jepsen M, Bauer M, Kessing LV (2018). Smartphone-based objective monitoring in bipolar disorder: status and considerations. Int J Bipolar Dis.

[CR12] Fisher AJ, Boswell JF (2016). Enhancing the personalization of psychotherapy with dynamic assessment and modeling. Assessment.

[CR13] Knapen SE (2019). Rhythm & Blues: Chronobiology in the Pathophysiology and Treatment of Mood Disorders.

[CR14] Kramer I, Simons CJP, Hartmann JA, Menne-Lothmann C, Viechtbauer W, Peeters F (2014). A therapeutic application of the experience sampling method in the treatment of depression: a randomized controlled trial. World Psychiatry.

[CR15] Larson R, Csikszentmihalyi M. The experience sampling method. New Directions for Methodology of Social & Behavioral Science. 1983.

[CR16] Lean M, Fornells-Ambrojo M, Milton A, Lloyd-Evans B, Harrison-Stewart B, Yesufu-Udechuku A (2019). Self-management interventions for people with severe mental illness: systematic review and meta-analysis. Br J Psychiatry.

[CR17] Leverich GS, Post RM. The NIMH life chart manual for recurrent affective illness: The LCM. NIMH Monograph. 1993.

[CR18] Michalak EE, Yatham LN, Kolesar S, Lam RW (2006). Bipolar disorder and quality of life: a patient-centered perspective. Qual Life Res.

[CR19] Miller CJ, Johnson SL, Eisner L (2009). Assessment tools for adult bipolar disorder. Clin Psychol Sci Pract.

[CR20] Murnane EL, Cosley D, Chang P, Guha S, Frank E, Gay G (2016). Self-monitoring practices, attitudes, and needs of individuals with bipolar disorder: implications for the design of technologies to manage mental health. J Am Med Inform Assoc.

[CR21] Murray G, Suto M, Hole R, Hale S, Amari E, Michalak EE (2011). Self-management strategies used by ‘high functioning’ individuals with bipolar disorder: from research to clinical practice. Clin Psychol Psychother..

[CR26] Myin-Germeys I, Mehl MR, Conner TS (2012). Psychiatry. Handbook of Research Methods for Studying Daily Life.

[CR22] Myin-Germeys I, Kasanova Z, Vaessen T, Vachon H, Kirtley O, Viechtbauer W (2018). Experience sampling methodology in mental health research: new insights and technical developments. World Psychiatry.

[CR23] O’Brien BC, Harris IB, Beckman TJ, Reed DA, Cook DA (2014). Standards for reporting qualitative research: a synthesis of recommendations. Acad Med.

[CR24] Perez Arribas I, Goodwin GM, Geddes JR, Lyons T, Saunders KE (2018). A signature-based machine learning model for distinguishing bipolar disorder and borderline personality disorder. Transl Psychiatry.

[CR25] Pini S, de Queiroz V, Pagnin D, Pezawas L, Angst J, Cassano GB (2005). Prevalence and burden of bipolar disorders in European countries. Eur Neuropsychopharmacol.

[CR27] Reis HT, Mehl MR, Conner TS (2012). Why researchers should think 'real-world': A conceptual rationale. Handbook of Research Methods for Studying Daily Life.

[CR28] Rush AJ, Trivedi MH, Ibrahim HM, Carmody TJ, Arnow B, Klein DN (2003). The 16-item Quick Inventory of Depressive Symptomatology (QIDS), clinician rating (QIDS-C), and self-report (QIDS-SR): A psychometric evaluation in patients with chronic major depression. BiolPsychiatry.

[CR29] Saunders KE, Bilderbeck AC, Panchal P, Atkinson LZ, Geddes J, Goodwin GM (2017). Experiences of remote mood and activity monitoring in bipolar disorder: a qualitative study. Eur Psychiatry.

[CR30] Schärer LO, Krienke UJ, Graf S-M, Meltzer K, Langosch JM (2015). Validation of life-charts documented with the personal life-chart app–a self-monitoring tool for bipolar disorder. BMC Psychiatry.

[CR31] Schermer M (2009). Telecare and self-management: opportunity to change the paradigm?. J Med Ethics.

[CR32] Smit AC, Snippe E, Wichers M (2019). Increasing restlessness signals impending increase in depressive symptoms more than 2 months before it happens in individual patients. Psychother Psychosom.

[CR33] Snippe E, Simons CJP, Hartmann JA, Menne-Lothmann C, Kramer I, Booij SH (2016). Change in daily life behaviors and depression: within-person and between-person associations. Health Psychol.

[CR34] Tsanas A, Saunders K, Bilderbeck A, Palmius N, Osipov M, Clifford G (2016). Daily longitudinal self-monitoring of mood variability in bipolar disorder and borderline personality disorder. J Affect Disord.

[CR35] Tyler E, Jones S, Black N, Carter L-A, Barrowclough C (2015). The relationship between bipolar disorder and cannabis use in daily life: an experience sampling study. PLoS ONE.

[CR36] Vachon H, Viechtbauer W, Rintala A, Myin-Germeys I (2019). Compliance and retention with the experience sampling method over the continuum of severe mental disorders: meta-analysis and recommendations. J Med Internet Res.

[CR37] van der Krieke L, Jeronimus BF, Blaauw FJ, Wanders RBK, Emerencia AC, Schenk HM (2016). HowNutsAreTheDutch ((HoeGekIsNL): A crowdsourcing study of mental symptoms and strengths. Int J Methods Psychiatr Res.

[CR38] van Genugten CR, Schuurmans J, Lamers F, Riese H, Penninx BW, Schoevers RA (2020). Experienced burden of and adherence to smartphone-based ecological momentary assessment in persons with affective disorders. J Clin Med.

[CR39] van Os J, Delespaul P, Wigman J, Myin-Germeys I, Wichers M (2013). Beyond DSM and ICD: introducing “precision diagnosis” for psychiatry using momentary assessment technology. World Psychiatry.

[CR40] van Os J, Verhagen S, Marsman A, Peeters F, Bak M, Marcelis M (2017). The experience sampling method as an mHealth tool to support self-monitoring, self-insight, and personalized health care in clinical practice. DepressAnxiety.

[CR41] Wenze SJ, Armey MF, Miller IW (2014). Feasibility and acceptability of a mobile intervention to improve treatment adherence in bipolar disorder: a pilot study. Behav Modif.

[CR42] Wenze SJ, Armey MF, Weinstock LM, Gaudiano BA, Miller IW (2016). An open trial of a smartphone-assisted, adjunctive intervention to improve treatment adherence in bipolar disorder. J Psychiatr Practice.

[CR43] Wichers M, Simons CJP, Kramer IMA, Hartmann JA, Lothmann C, Myin-Germeys I (2011). Momentary assessment technology as a tool to help patients with depression help themselves. Acta Psychiatr Scand.

[CR44] Wichers M, Snippe E, Riese H, Bos FM (2019). The network approach to depression: Hype or Holy Grail?. Gedragstherapie.

