# Supplementary material for: Recommendations for the use of long-term experience sampling in bipolar disorder care: a qualitative study of patient and clinician experiences
Source: Int J Bipolar Disord. 2020 Dec 1;8:38. doi: 10.1186/s40345-020-00201-5 (PMC7704990; doi:10.1186/s40345-020-00201-5)
Supplement: Supplementary file 1 — Additional file 1. ESM items, data security, and example of feedback report. [file 40345_2020_201_MOESM1_ESM.docx]

Supplementary materials for:

Recommendations for the use of long-term experience sampling in bipolar disorder care: A qualitative study of patient and clinician experiences

Fionneke M. Bos*^1,2^, M.Sc.

Evelien Snippe^2^, PhD

Richard Bruggeman^1^, MD PhD

Bennard Doornbos^3^, MD PhD

Marieke Wichers^2^, PhD

Lian van der Krieke^1,2^, PhD

^1^ University of Groningen, University Medical Center Groningen, Rob Giel Research Center, Department of Psychiatry, Groningen, The Netherlands

^2^ University of Groningen, University Medical Center Groningen, Interdisciplinary Center Psychopathology and Emotion Regulation (ICPE), Department of Psychiatry, Groningen, The Netherlands

^3^ Department of Specialized Training, Psychiatric Hospital Mental Health Services Drenthe, Outpatient Clinics, Assen, The Netherlands

*Corresponding author:

Fionneke Bos, M.Sc., Rob Giel Research Center, University of Groningen, University Medical Center Groningen, PO Box 30.001, 9700 RB, Groningen, The Netherlands. Phone: +31 50 361 5725, e-mail: [f.m.bos01@umcg.nl](mailto:f.m.bos01@umcg.nl).

| Supplementary Table 1. ESM diary items. | | | | |
| --- | --- | --- | --- | --- |
|  | **Dutch** | **English translation** | **Anchors (far left, middle, far right)** | **Which pompt(s)** |
|  | Is dit de eerste meting die u invult vandaag? | Is this the first assessment you complete today? | - Yes - No | All five prompts |
|  | 1. Hoe lang heeft u geslapen? | How long did you sleep? | 0 – 12 hours | If answered ‘yes’ on item 1 |
|  | 1. De kwaliteit van mijn slaap was… | The quality of my sleep was… | Very bad – reasonably – very good | If answered ‘yes’ on item 1 |
|  | Ik voel me opgewekt | I feel cheerful | Not at all – reasonably – very much | All five prompts |
|  | Ik voel me neerslachtig | I feel down | Not at all – reasonably – very much | All five prompts |
|  | Ik voel me kalm | I feel calm | Not at all – reasonably – very much | All five prompts |
|  | Ik voel me gejaagd | I feel agitated | Not at all – reasonably – very much | All five prompts |
|  | Ik voel me bijzonder goed | I feel extremely well | Not at all – reasonably – very much | All five prompts |
|  | Ik voel me moe | I feel tired | Not at all – reasonably – very much | All five prompts |
|  | Ik voel me tevreden | I feel content | Not at all – reasonably – very much | All five prompts |
|  | Ik voel me geïrriteerd | I feel irritated | Not at all – reasonably – very much | All five prompts |
|  | Ik zit vol energie | I am full of energy | Not at all – reasonably – very much | All five prompts |
|  | Ik zie op tegen de rest van de dag | I dread the rest of the day | Not at all – reasonably – very much | All five prompts |
|  | Ik zit vol goede ideeën | I am full of good ideas | Not at all – reasonably – very much | All five prompts |
|  | Ik heb het gevoel te kort te schieten | I feel inadequate | Not at all – reasonably – very much | All five prompts |
|  | Mijn gedachten gaan snel | My thoughts are racing | Not at all – reasonably – very much | All five prompts |
|  | Ik kan snel schakelen | I am able to focus and switch easily | Not at all – reasonably – very much | All five prompts |
|  | Ik ben snel afgeleid | I’m distracted easily | Not at all – reasonably – very much | All five prompts |
|  | Ik heb zin om met anderen af te spreken | I feel like socializing | Not at all – reasonably – very much | All five prompts |
|  | Eigen vraag | Personal question | Not at all – reasonably – very much | All five prompts |
|  | Ik heb meer gegeten dan gewoonlijk | I have eaten more than usual | Not at all – reasonably – a lot more than usual | All five prompts |
|  | Sinds het vorige meetmoment heb ik gepiekerd | Since the previous prompt, I have worried | Not at all – reasonably – very much | All five prompts |
|  | Sinds het vorige meetmoment heb ik veel gecommuniceerd | Since the previous prompt, I have communicated a lot | Not at all – reasonably – very much | All five prompts |
|  | Sinds het vorige meetmoment heb ik me lichamelijk ingespannen | Since the previous prompt, I have been physically active | Not at all – reasonably – very much | All five prompts |
|  | Denk aan de meest opvallende gebeurtenis sinds het vorige meetmoment. Hoe heftig was deze gebeurtenis? | Think back on the most notable event since the previous prompt. How intense was this event? | Not at all – reasonably – very much | All five prompts |
|  | Is dit de laatste meting die u invult vandaag? | Is this the last assessment you complete today? | - Yes - No | All five prompts |
|  | 1. Ik heb vandaag veel afspraken gemaakt | I have made a lot of appointments today | Not at all – reasonably – a lot | If answered ‘yes’ on item 26 |
|  | Sinds het vorige meetmoment, wat deed ik? | Since the previous prompt, what have I been doing? (multiple options possible) | - Sleeping  - Household chores/groceries  - Working/studying  - Doing sports/walking/cycling  - Something relaxed (e.g., reading, TV)  - Hobby (e.g., gardening, music)  - A trip (e.g., into town, concert)  - Something together with others  - Something intimate (e.g., cuddling, sex)  - Engaging in self-care  - Resting/nothing  - On the way  - Something else | All five prompts |
|  | Noteer hier eventuele opmerkingen. Noteer het ook als er iets gebeurd is dat invloed heeft op uw stemming. | Note observations here if any. Also note anything that may have influenced your mood. | Open entry | All five prompts (optional) |

1. **Experience sampling method diary items**

The item list was constructed in several steps. First, we identified relevant concepts for symptoms of bipolar disorder and searched the literature for ESM studies in patients with bipolar disorder. This yielded a first item list of 71 potentially relevant items. This list was then discussed in individual interviews with three patients and one psychiatrist. The items were finally selected on three criteria: 1) the patients and clinician recognized the item as signaling symptoms of either mania or depression; 2) the patients and the clinician felt comfortable with the formulation of the item (e.g., they could see themselves saying the sentence in daily life); 3) both the patients, the clinician, and the research team believed the item would vary meaningfully within participants. Items that were deemed relevant but too person-specific were put on a list for participants so they might select them for their personal question.

All items were obligatory, with the exception of the comment field at the end of the questionnaire (item 29). All items were assessed five times per day, with the exceptions of the items regarding sleep (item 2- 3), and the item regarding appointments (item 27). These items were only shown if participants answered ‘yes’ on item 1 or item 26. This way, we ensured that participants could still answer questions about their sleep, even though they might have skipped the first (few) assessments.

1. **Data security**

ESM assessments, as well as the weekly ASRM and QIDS questionnaires, were administered and secured via RoQua ([www.roqua.nl](http://www.roqua.nl)). RoQua is a web-based application that is fully integrated in the personal health record systems used by several mental health care institutions in the Northern Netherlands that are part of the Rob Giel Research Center ([www.rgoc.nl](http://www.rgoc.nl)). The two institutions that participated in the present study are part of this collective and use the RoQua application for Routine Outcome Monitoring (ROM).

RoQua links all assessment data to a personal identifier, removing all information that may be traceable to the individual, thereby ensuring that the data is stored anonymously and securely. Given that RoQua is used in clinical practice, the application meets the stringent criteria regarding privacy and data security. During the study period, only the patients’ clinicians and the researcher FB could access the patients’ assessment data. Patients gave consent to both their clinicians and researcher FB to view the ESM and weekly questionnaire data.

1. **Personal feedback report**

The personal feedback report consisted mostly of descriptive information. Below, we provide an overview of the feedback that was given to each participant, based on simulated data. This way, we are able to show all graphs and explanations offered to participants, as well as explain our data analytic choices herein.

All feedback reports contained graphs, as well as text to explain how the graphs should be read. The report did not give advice on the interpretation of the graphs (e.g., ‘this graph shows exercising is good for your health’); this was left to the patient and clinician to decide.

**Missing data.** First, participants were shown their percentage of missing data across all the five time points in a bar plot (see Figure 1). They were presented with their overall percentage of completed assessments (e.g., 76%), and whether sufficient assessments were completed to give reliable information about their data.

Figure 1. Percentage of completed assessments at each of the assessment times.


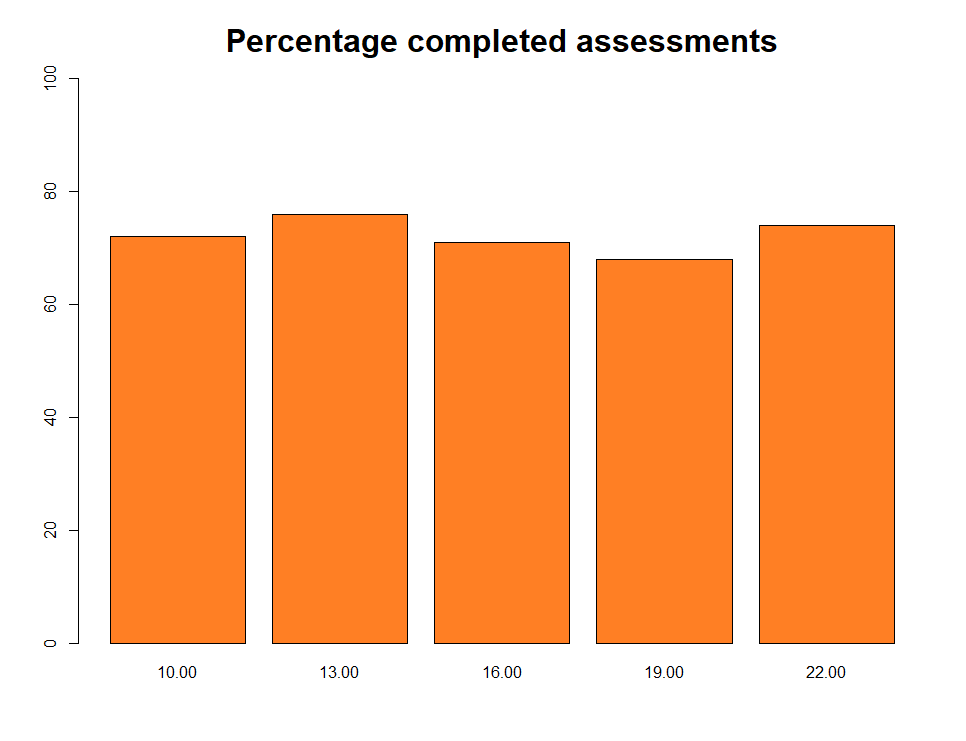


**Time of day.** Next, mood and symptoms in relation to time of day was depicted in bar plots with error bars (see figure 2). Participants were explained that, when the error bars did *not* overlap one another, there was, on average, a difference in how they felt over the course of the day. These plots were shown for every diary item that significantly differed across the five time points. If there were significant differences, participants were told how their symptoms varied (e.g., ‘during the monitoring period, you generally felt more down in the morning than in the afternoon and evening’). If no significant differences arose, no plots were shown, and participants were explained that we could not find indications of diurnal variation in mood and symptoms.


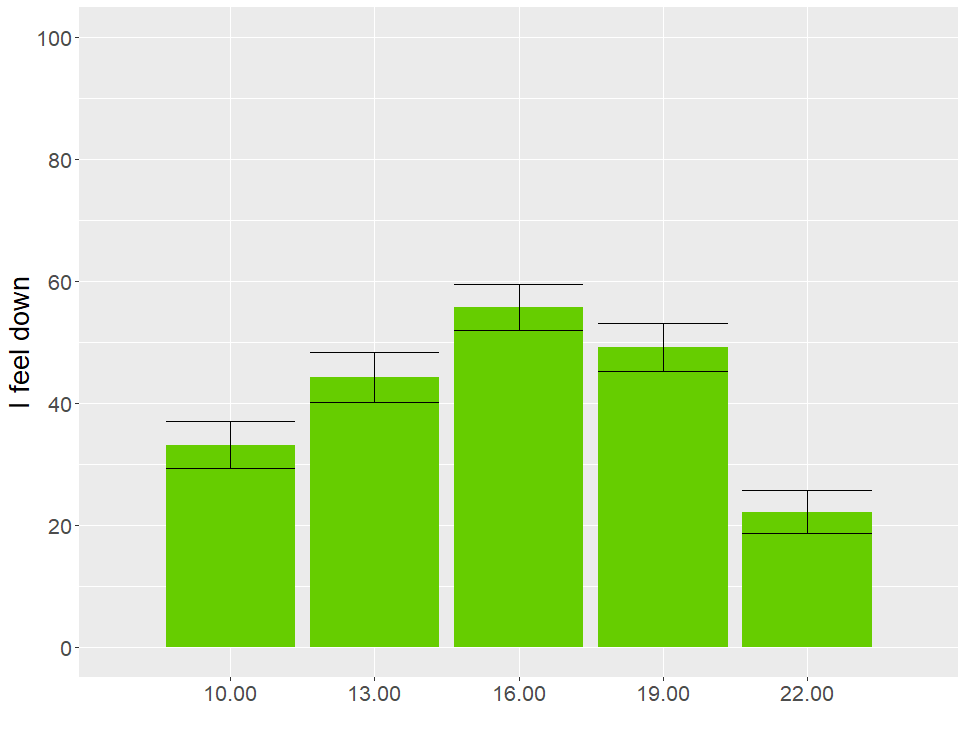
Figure 2. Diurnal variation in the item “I feel down”.

**Activities.** Furthermore, participants were shown the percentage of time they spent on different activities and their average mood during these activities (cheerful and down) in bar plots (see Figure 3). Participants were explained that they could not draw causal inferences from these plots, mainly due to two limitations. First, during the monitoring period, participants had retrospectively indicated every activity they had done during the previous three hours. This means that the relationship between mood and a particular activity could be confounded by function of time (e.g., the activity was done at the beginning of the three hour time block but not at the time of the assessment, when mood was assessed). Second, the relationships between mood and activities was bidirectional. A certain mood could influence the likelihood of an activity, whereas activities could also induce a certain mood.


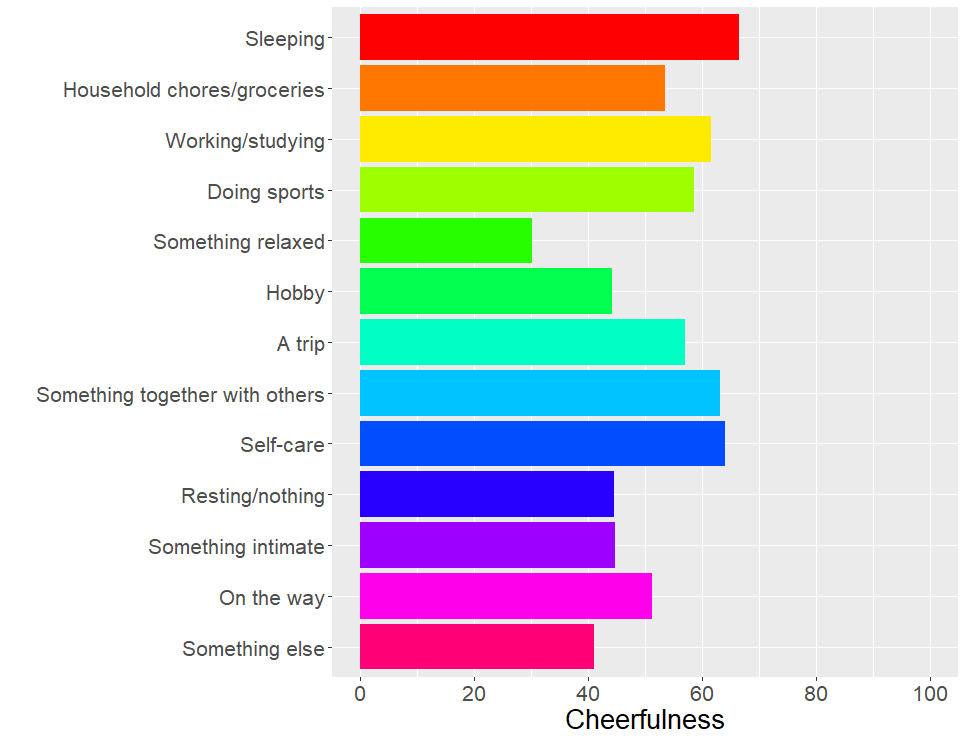

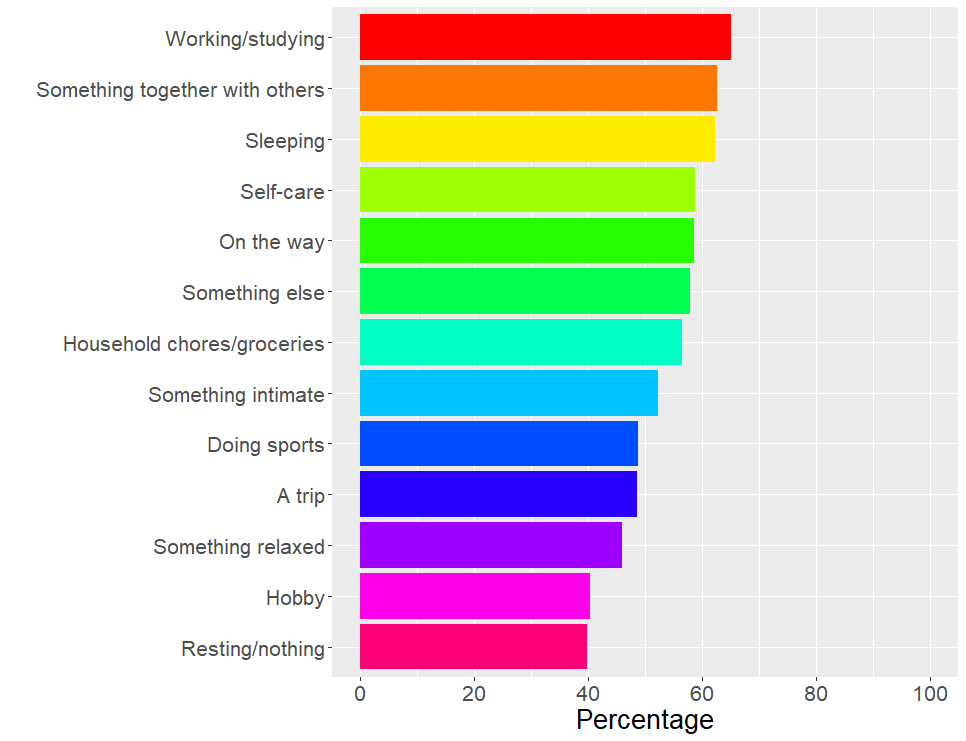
Figure 3. Percentage of time spent on activities and mood during activities.

**Episode occurrence.** Then, participants were shown the weekly mood questionnaires (QIDS and ASRM) in line graph (see Figure 4). Participants were explained that previous research [1-4] had established cut-offs (see the orange and red lines), above which there could be indications of a (hypo)manic or depressive episode.


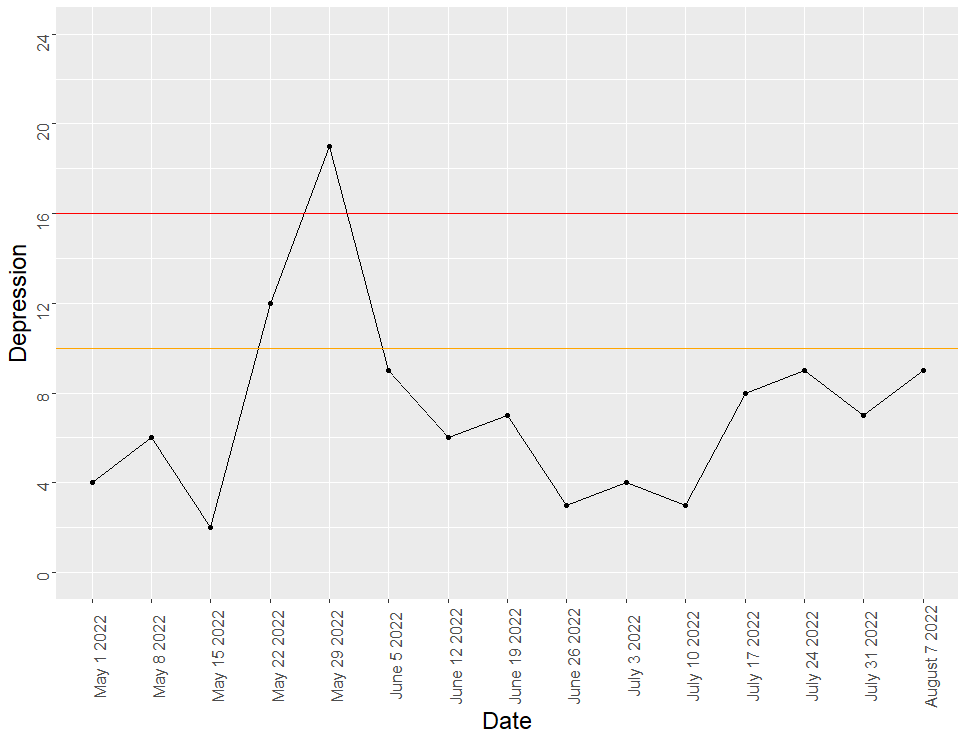

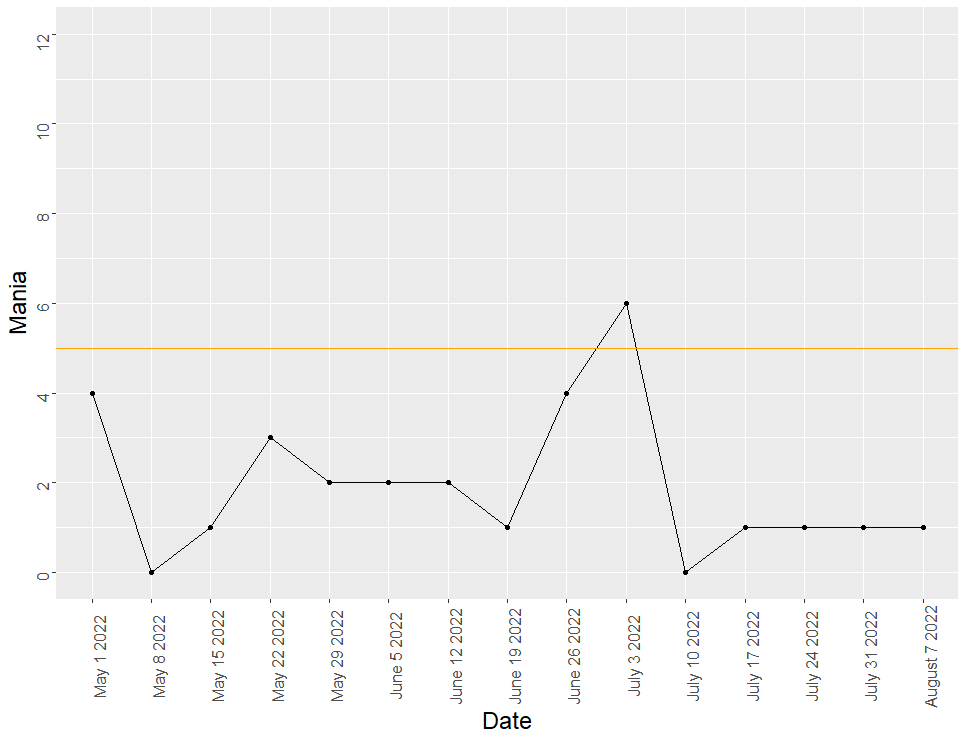
Figure 4. Weekly scores on manic (ASRM) and depressive (QIDS) symptoms.

Possible severe depression

Possible depression

Possible (hypo)mania

**Sleep and appointments.** Next, line graphs depicting the once per day variables (having made appointments, sleep duration, and sleep quality) were shown (Figure 5). Vertical lines depicted potential episodes of (hypo)mania and depression, to visualize potential associations between the weekly questionnaires and the ESM items. Given that the items of the weekly questionnaires pertained to the previous week, we highlighted both the day of completing the questionnaire (bold blue/red line) and the previous week (transparent bar). Missing data was depicted as a broken line.


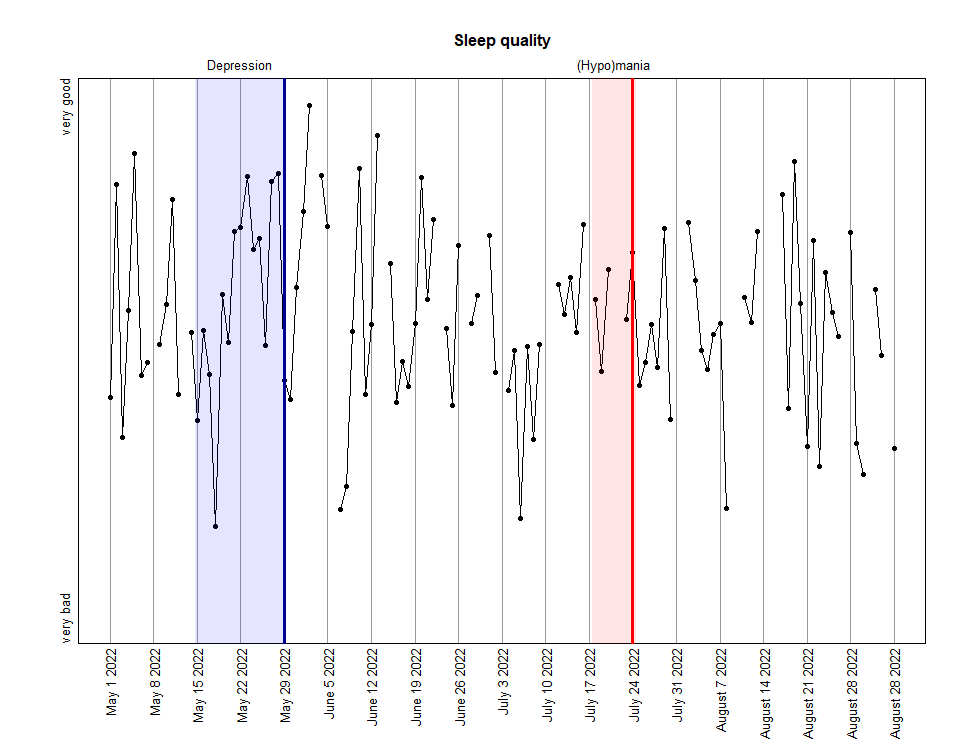
Figure 5. Variation in sleep quality, plotted against weeks with increased (hypo)manic and depressive symptoms.

**Mood and symptom variation.** Similar plots were constructed for all other continuous ESM variables (22 in total; see Figure 6). To facilitate visual inspection of the graphs, we fitted Kernel smoothing lines over the plots. This helped patients and clinicians to view weekly trends in the ESM data (e.g., has this item decreased or increased in a particular week).


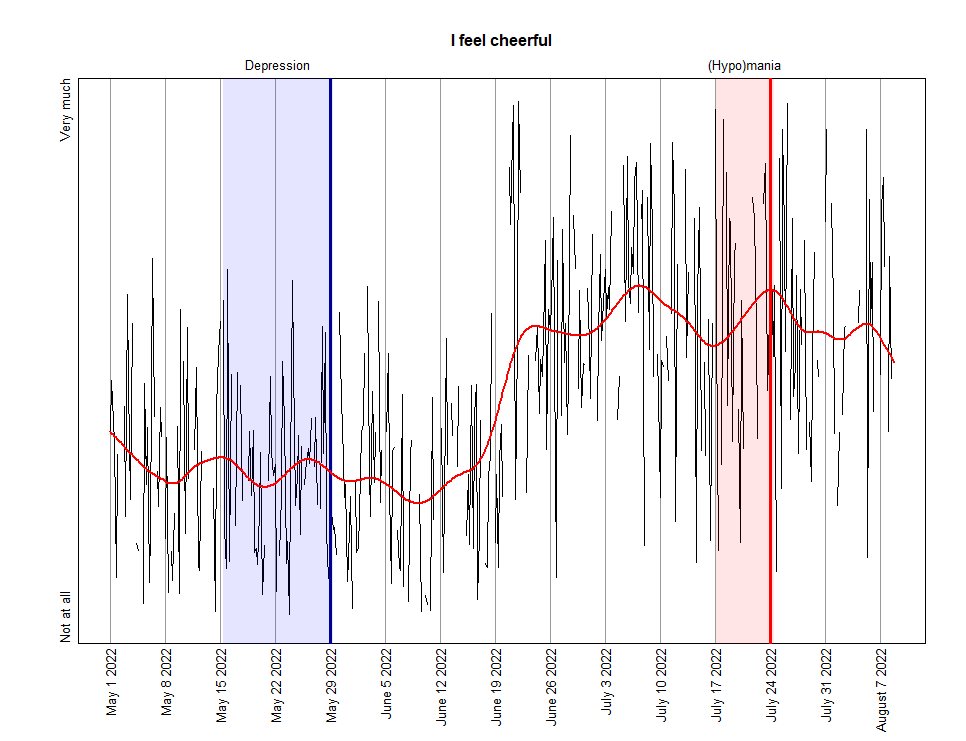
Figure 6. Mood variation for the item “I feel cheerful”.

**Episode indicators.** To facilitate interpretation of all 22 line graphs, the researcher examined the Kernel smoothing lines of each graph to determine which variables uniquely increased or decreased during (hypo)manic or depressive episodes (and not during euthymic periods). When such unique variables were found, these were summarized in another line graph (see Figure 7). Participants were explained that they might monitor themselves for these variables specifically to recognize impending episodes. If no indicator variables were found, participants were explained that we could not find specific variables that might signal a depression or (hypo)mania.

Figure 7. Indicator plot for (hypo)mania.


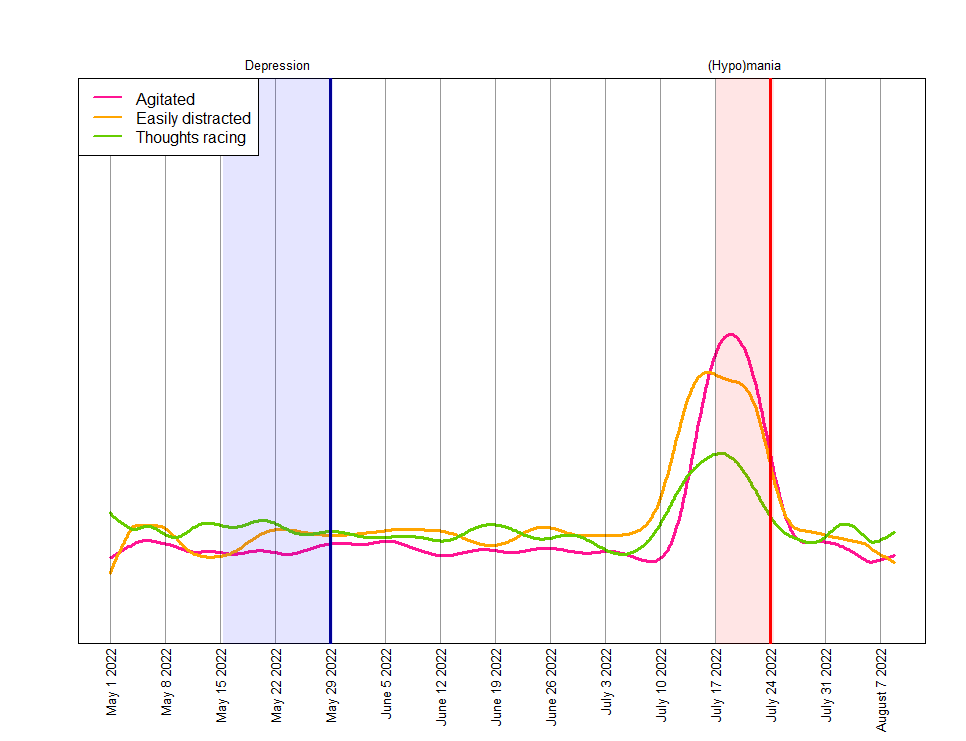


**Associations between variables (optional).** Some participants wished to see information on the associations between specific variables (e.g., between mood and physical activity or time spent with loved ones). This was tested using lag-1 vector autoregressive models (VAR [5]) through the AutovarCore package [6]. This R-package automatically estimates individual VAR-models. A VAR-model was only constructed if 1) the participant had completed at least 60% of all assessments, 2) the items of interest showed sufficient variation (>10% [7]), and 3) the items were stationary. Results were only deemed significant if all four autovarCore models were statistically significant. Together with the participant, we operationalized the research question, thereby attempting to minimize the number of variables so as to reduce the risk of Type I error.

If a significant association was found, participants were told that we found indications that, in general, variable A resulted in less/more variable B three hours later (e,g, being more physically active led to feeling more cheerful three hours later; see Figure 8). To caution overinterpretation, we stated that this does not necessarily mean that this is always the case, and that these results might be specific to the monitoring period.

If no significant associations were found, participants were told that we could not find indications that variable A significantly influenced variable B three hours later or vice versa. Participants were explained that this did not mean that the association could never exist, but that this could be due to the constraints of the data and the model (e.g., exercising could have a beneficial effect on other variables that were not tested, or exercising could have a beneficial effect at other lags than lag-1).

Figure 8. Association between being physically active and cheerfulness. This graph shows that, if the participant was physically active, he/she would generally feel more cheerful three hours later.

**MORE**

**Comment field.** Finally, participants were presented with a table containing all their time-stamped responses to the comment field (see Figure 9). Patients and clinicians were encouraged to use them to interpret peaks and valleys in the line graphs.

Figure 9. Table containing (fictional) responses in the comment field.

| Date | Diary entry |
| --- | --- |
| 12-7-2022 9:47 | e.g. talked to person A about X. |
| 13-7-2022 13:01 | e.g. headache |
| 13-7-2022 21:46 | e.g. felt down because of Y. |
|  |  |

References

1. Rush, A.J., et al., *The 16-item Quick Inventory of Depressive Symptomatology (QIDS), clinician rating (QIDS-C), and self-report (QIDS-SR): A psychometric evaluation in patients with chronic major depression.* Biological psychiatry, 2003. **54**(5): p. 573.

2. Altman, E.G., et al., *The Altman self-rating mania scale.* Biological psychiatry, 1997. **42**(10): p. 948.

3. Altman, E., et al., *A comparative evaluation of three self-rating scales for acute mania.* Biological psychiatry, 2001. **50**(6): p. 468.

4. Miller, C.J., S.L. Johnson, and L. Eisner, *Assessment tools for adult bipolar disorder.* Clinical Psychology: Science and Practice, 2009. **16**(2): p. 188-201.

5. Brandt, P. and J.T. Williams, *Multiple time series models*. 2007, Thousand Oaks, CA, US: Sage Publications Inc.

6. Emerencia, A.C., et al., *Automating Vector Autoregression on Electronic Patient Diary Data.* IEEE Journal Of Biomedical And Health Informatics, 2016. **20**(2): p. 631.

7. Brose, A. and N. Ram, *Within-person factor analysis: Modeling how the individual fluctuates and changes across time*, in *Handbook of Research Methods for Studying Daily Life*, M.R. Mehl and T.S. Conner, Editors. 2012, Guilford Press: New York, NY US. p. 459.
